# A Novel Deformity Correction Manipulation System for Better Correction of Large Thoracic Scoliosis

**DOI:** 10.1111/os.14169

**Published:** 2024-07-27

**Authors:** Yong Huang, Ce Zhu, Yongliang Wang, Ganjun Feng, Limin Liu

**Affiliations:** ^1^ Department of Orthopedic Surgery and Orthopedic Research Institute, West China Hospital Sichuan University Chengdu China; ^2^ Department of Orthopedic Surgery Pangang Group General Hospital Panzhihua China

**Keywords:** Correction technique, Deformity correction manipulation system, Thoracic scoliosis

## Abstract

**Objective:**

Treating patients with large thoracic scoliosis (between 70° and 100°) poses technical challenges, particularly with traditional correction techniques (TCT). To address this, we developed a novel deformity correction manipulation system (DCMS) aimed at reducing surgical complexity and trauma. This study aims to assess the safety and effectiveness of DCMS in treating large thoracic scoliosis.

**Methods:**

From January 2016 to June 2021, 76 patients with large thoracic scoliosis were included in this retrospective study. The patients were divided into two groups: DCMS (*n* = 34) and TCT (*n* = 42). Basic patient data including age at surgery, sex, etiology, Risser sign, flexibility of the main thoracic curve, instrumented levels, number of screws, duration of hospital stay, and follow‐up time were collected and analyzed. Radiographic and clinical outcomes, as measured by various radiographic parameters and Scoliosis Research Society‐30 (SRS‐30) scores, were retrospectively analyzed and compared between the two groups. Adverse events were also documented. Statistical analyses were performed using two‐tailed independent *t*‐tests, chi‐square tests, and Fisher's exact test.

**Results:**

The DCMS group exhibited significantly shorter operative times, reduced blood loss, and shorter hospital stays compared to the TCT group. However, there were no significant differences between the two groups in terms of age at surgery, sex, etiology, Risser sign, flexibility of the main curve, instrumented levels, number of screws, and follow‐up time. While preoperative major curves were statistically similar between the two groups, the DCMS group achieved a superior correction rate compared to the TCT group (74.2% ± 8.8% *vs* 68.1% ± 10.5%). No significant differences were observed in other radiographic parameters, SRS‐30 scores, or the incidence of adverse events.

**Conclusion:**

The application of DCMS resulted in shorter operative times, reduced blood loss, shorter hospital stays, and greater curve correction compared to TCT. DCMS proves to be a safe and effective technique for treating large thoracic curves.

## Graphical abstract

Deformity correction manipulation system (DCMS). (A) Multiple‐screw linkage distraction reducer (MLDR) system: cranial and caudal manipulating arms are designed to perform distraction force and the middle cantilever manipulating arms with threaded shaft are designed to perform lifting force for derotation. Together they are connected to a coupling rod by couplers. The manipulating arms are attached to proximal, apical, and distal regions of the spine via three provisional rods. (B). Manipulating handle couples with the hexagonal connector to regulate the manipulating arms. (C) Schematic diagram of the MLDR system. (D) In situ cantilever bending (ISCB) system: traditional two‐rod bending devices were attached to the spine. Two adjustable and removable clips were connected to the lever arms of the rod‐bending device. The anterior clip is designed to stabilize the system and the posterior clip is designed to modulate the degree of compression. (E) Schematic diagram of the ISCB system.

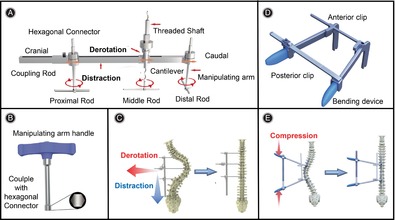

## Introduction

Significant advancements have occurred in the treatment of thoracic scoliosis in recent decades. Posterior spinal fusion has emerged as a relatively secure procedure, offering comprehensive deformity correction while circumventing the negative impacts associated with anterior approaches.^1^, ^2^ Traditional correction techniques (TCT), including derotation of the concave rod, direct vertebral rotation, bilateral apical vertebral derotation, and differential rod contouring, have become integral to this approach, establishing it as the primary surgical method for spinal scoliosis. TCT demonstrates notable efficacy in restoring coronal and sagittal balance while facilitating vertebral de‐rotation in the transverse plane.^3^, ^4^, ^5^, ^6^ A growing body of evidence supports the efficacy and safety of posterior spinal fusion with TCT in mild to moderate thoracic scoliosis.^7^, ^8^, ^9^

Recent literature suggests that posterior‐only approaches may yield comparable correction rates to anteroposterior approaches, even in cases of large and stiff scoliosis.^10^, ^11^, ^12^ Despite these promising findings, posterior spinal fusion with traditional correction techniques (TCT) for large curves presents several challenges. First, addressing large scoliosis necessitates a demanding surgical procedure that requires heightened skill and time, and poses a significant risk of complications. Second, applying sufficient correction force becomes increasingly challenging with existing TCT as curves grow larger and stiffer. Lastly, significant friction arises in large scoliosis cases between rods and screws, particularly at the periapical vertebrae. This friction impedes the movement of the concave rod during deformity correction and can lead to screw plow or pull‐out during translation and de‐rotation of the scoliotic spine.^13^, ^14^ Consequently, larger curves are associated with prolonged operative times, greater intraoperative blood loss, higher costs, and delayed return to function, as indicated by previous research.^15^, ^16^ Thus, achieving satisfactory correction with minimal damage remains a significant challenge, particularly in cases of large thoracic scoliosis (between 70° and 100°).

**FIGURE 1:**
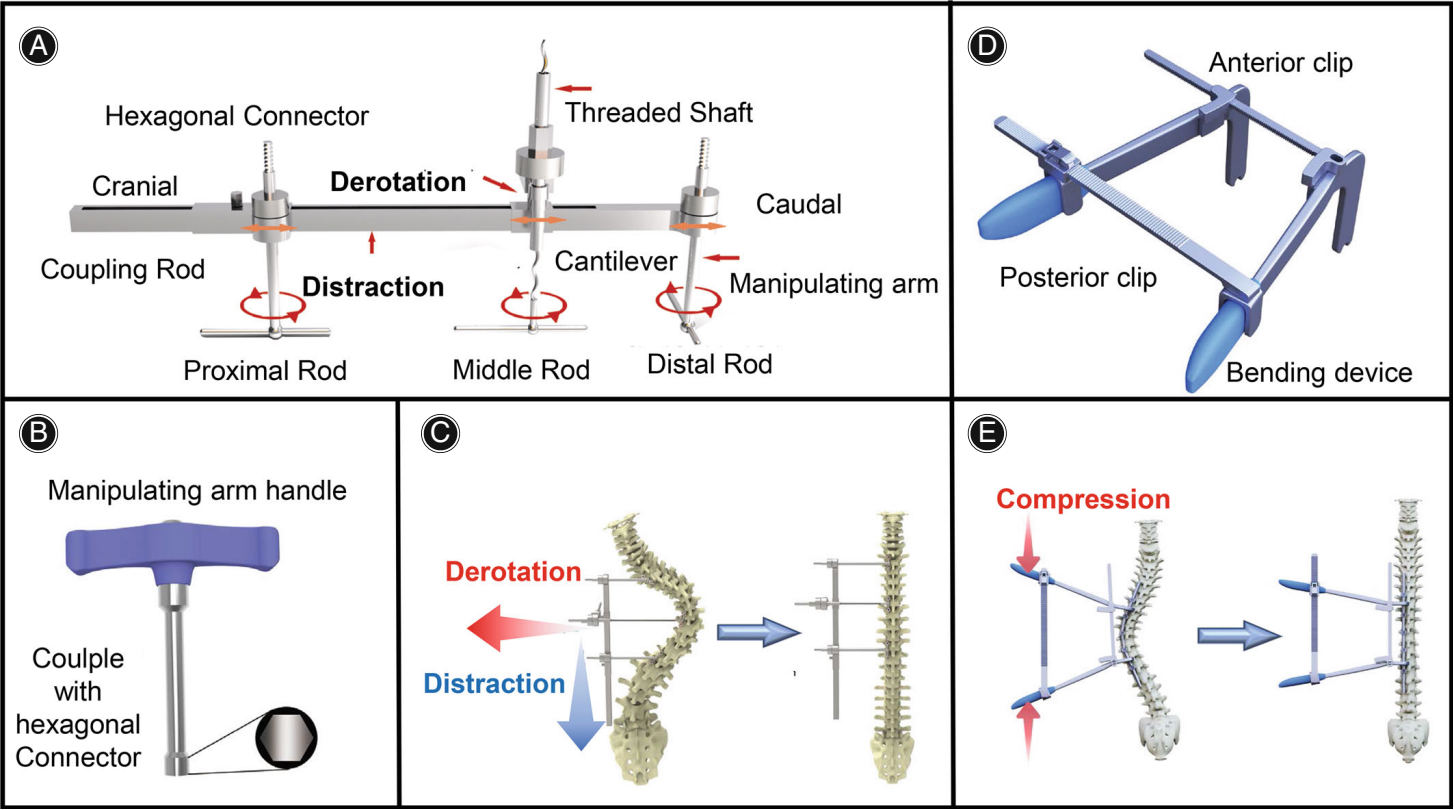
Deformity correction manipulation system (DCMS). (A) Multiple‐screw linkage distraction reducer (MLDR) system: cranial and caudal manipulating arms are designed to perform distraction force and the middle cantilever manipulating arms with threaded shaft is designed to perform lifting force for derotation. Together they are connected to a coupling rod by couplers. The manipulating arms are attached to proximal, apical, and distal regions of the spine via three provisional rods. (B). Manipulating handle couples with the hexagonal connector to regulate the manipulating arms. (C) Schematic diagram of the MLDR system. (D) In situ cantilever bending (ISCB) system: Traditional two‐rod bending devices were attached to the spine. Two adjustable and removable clips were connected to the lever arms of the rod‐bending device. The anterior clip is designed to stabilize the system and the posterior clip is designed to modulate the degree of compression. (E) Schematic diagram of the ISCB system.

In pursuit of a solution, we have developed a novel deformity correction manipulation system (DCMS) comprised of a multiple‐screw linkage distraction reducer (MLDR) and in situ cantilever bending (ISCB) (Figure 1). We hypothesized that the innovative system would provide faster, simpler, and more effective correction for the treatment of large thoracic scoliosis. Therefore, this study aimed to investigate the following objectives: (i) elucidate the design principles and advantages of DCMS; (ii) evaluate the efficacy of DCMS compared to TCT in the treatment of large thoracic scoliosis; and (iii) assess the safety of DCMS in the treatment of large thoracic scoliosis.

## Methods

### Study Design and Population

This study, approved by the local ethics committee (No. 2019852) and conducted with informed consent from patients and/or their parents, was a non‐randomized retrospective investigation between January 2016 and June 2021. The inclusion criteria were as follows: (i) a main thoracic curve between 70° and 100°; (ii) patients treated with either DCMS or TCT using one‐stage posterior spinal fusion surgery (PSF); and (iii) a minimum follow‐up period of 2 years. The exclusion criteria were: (i) Cobb angles <70° or > 100°; (ii) patients who underwent preoperative halo traction, anterior release, or staged growth rod distraction; (iii) congenital scoliosis; and (iv) incomplete follow‐up information. Ultimately, a total of 76 patients were included in this study, with 34 patients in the DCMS group and 42 patients in the TCT group.

### Data Collection

Preoperative data including the patient's sex, age at surgery, Risser sign, and etiology were collected. Operative data including instrumented levels, number of screws, duration of surgery, and surgical blood loss were collected. Postoperative data including duration of hospital stay, blood transfusion, adverse events, and follow‐up time were collected. Whole‐spine standing posteroanterior and lateral radiographs and Scoliosis Research Society‐30 (SRS‐30) questionnaire were reviewed preoperatively, 3 months postoperatively, and at the most recent follow‐up. Preoperative magnetic resonance imaging of the spine was reviewed to clarify the diagnosis of thoracic scoliosis.

### Radiographic Measurements and Clinical Results

**FIGURE 2:**
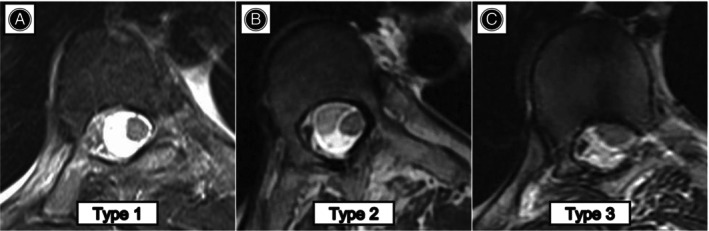
Representative images of spinal cord classification based on MRI.

All spinal radiographs were measured digitally by the synapse analysis system. Cobb angle obtained from supine maximum lateral bending films was used to calculate the flexibility. Radiographic data included the main curve, thoracic kyphosis (T5‐T12), lumbar lordosis (L1‐S1), apical vertebral translation, and coronal and sagittal imbalance. The distance between the C7 plumb line and the central sacral vertical line indicates coronal imbalance. The sagittal imbalance was indicated as the distance between the C7 plumb line and the posterosuperior corner of S1. Radiological parameters were measured at the following time points: preoperatively, 3 months postoperatively, and at the final follow‐up. The spinal cord MRI classification as described by Sielatycki *et al*. was collected.^17^ As shown in Figure 2, three types of cords were proposed: Type 1 which is a circular cord with visible cerebrospinal fluid; Type 2 which is a circular cord but no visible cerebrospinal fluid at apical concavity; and Type 3 which is a deformed cord with no intervening cerebrospinal fluid.

SRS‐30 questionnaires were completed and collected from all patients. There are five domains including pain, function, self‐image, mental health, and satisfaction. The average scores of each domain as well as the overall mean score were calculated at the final follow‐up time points.

### Surgical Techniques

**FIGURE 3:**
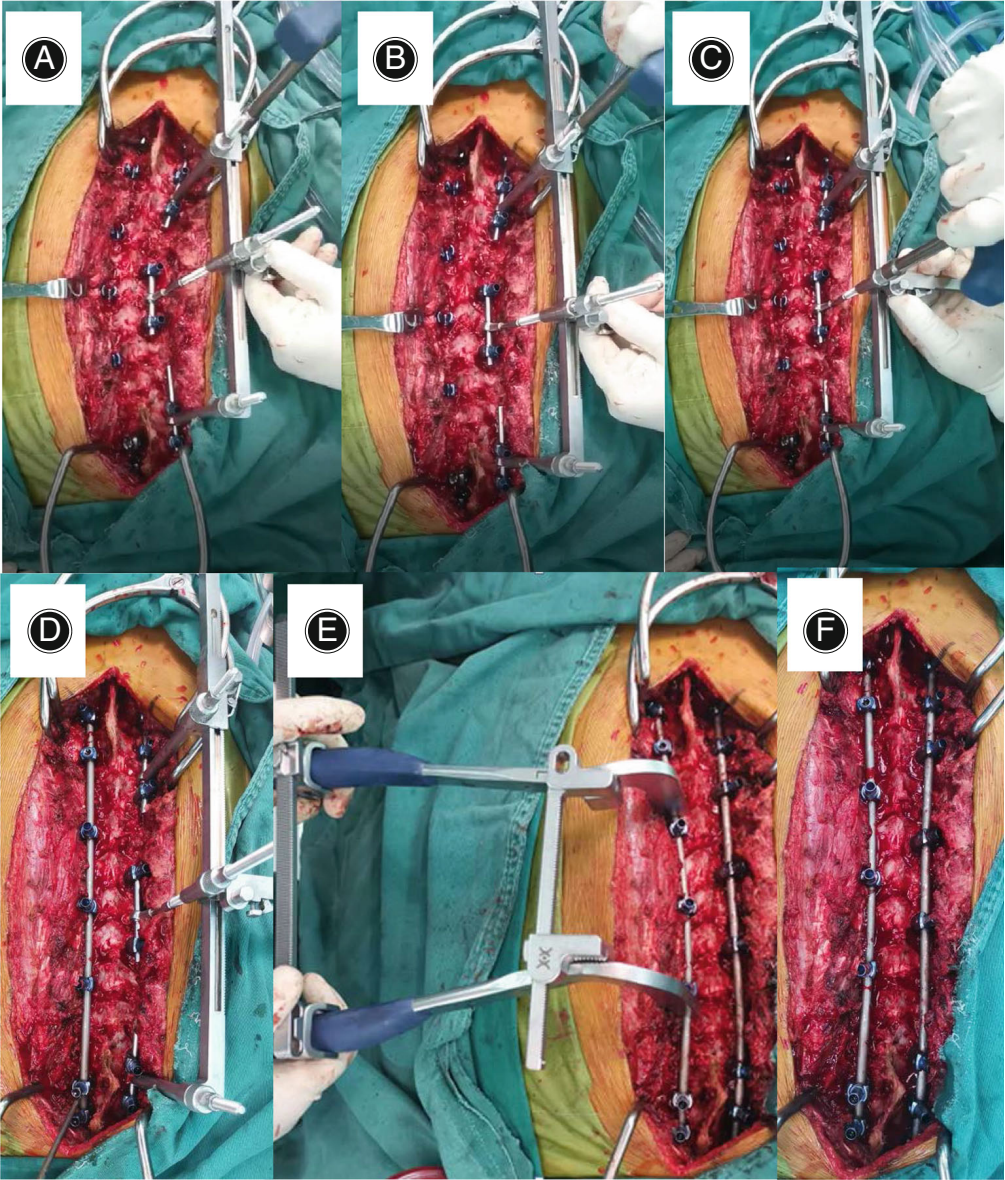
Procedures of DCMS technique: (A) MLDR devices were connected are attached to the proximal, apical, and distal segments on the concave side of the spine *via* provisional rods. (B) Distraction was performed by modulating the proximal and distal manipulating arms. (C) Decoration with lifting force was performed by modulating the cantilever manipulating arms. (D) Pre‐contoured permanent rod was prepared for the convex side. (E) ISCB is attached to the rod and provides in situ consistent compression force to further correct the coronal and rotational deformities. (F) The convex permanent rod is fastened with locking caps and maintains the deformity correction under the assistance of ISCB. MLDR is removed and another permanent rod is prepared and inserted into the concave side of the spine.

The design principles and structural characteristics of DCMS (Product code:08.301.02.23153000, WEGO Holding, Weihai, China) have been depicted in Figure 1. DCMS is utilized following exposure of the posterior elements of the scoliotic spine, wherein non‐continuous pedicle screws are inserted. Occasionally, multiple Smith–Petersen osteotomies or Ponte osteotomies are required to release the spine. The process unfolds as follows: initially, the MLDR is affixed to the concave side of the spine using three provisional rods, each secured separately in the proximal, apical, and distal segments *via* tightening set screws (Figure 3A). Provisional rods may necessitate contouring to align with the spinal curvature. Subsequently, the distraction manipulating arm of the MLDR is connected over the proximal and distal provisional rods (Figure 3B), providing consistent distraction force to correct the coronal deformity. Additionally, a threaded shaft with a cantilever lift device of the MLDR is linked to the apical provisional rod, aiding axial derotation of the spine and rib cage, and sagittal deformity correction by applying a persistent lift force (Figure 3C). Adjustment of the correction force is facilitated by rotating a hexagonal connector, ensuring precise control *via* a unidirectional sawtooth fixation design. Next, a pre‐contoured permanent rod is readied for the convex side once sufficient correction is attained on the concave side (Figure 3D). The ISCB is attached to the rod, supplying in situ consistent compression force to further rectify coronal and rotational deformities (Figure 3E). Subsequently, the convex permanent rod is secured with locking caps, maintaining the deformity correction with ISCB's assistance. Following removal of the MLDR, another permanent rod is prepared and inserted into the concave side of the spine (Figure 3F). Overcontouring the concave rod aids in further restoration of the sagittal plane. Any necessary compression, distraction, or in situ bending is performed before the final tightening of the construct. The videos of DCMS procedure were provided in supplementary files.

In the TCT group, scoliosis correction was achieved using traditional techniques involving rod derotation, translation, and direct vertebral derotation, as previously described.^18^ Intraoperative neuromonitoring, encompassing assessment of somatosensory evoked potentials (SSEPs) and motor evoked potentials (MEPs), was applied during all operations.

### Statistical Analysis

Data analysis was conducted utilizing IBM SPSS Statistics (IBM, Armonk, NY, USA). Continuous variables are presented as mean ± standard deviation, while categorical variables are expressed as frequencies. Two‐tailed independent *t*‐tests were employed to compare continuous variables between the two groups. For categorical variables, a chi‐square test was utilized. In cases of small sample sizes, a Fisher exact test was also considered. Statistical significance was defined as a *p*‐value <0.05.

## Results

### Demographic and Surgical Variables

In the DCMS group, there were eight male and 26 female patients, while the TCT group comprised 10 male and 32 female patients. Statistical analysis using *t*‐tests revealed that differences in age at surgery, sex, etiology, spinal cord classification, Risser sign, flexibility of the main curve, instrumented levels, number of screws, and follow‐up time were not statistically significant (*p* > 0.05). However, notable distinctions were observed in the mean duration of surgery (*p* < 0.001), estimated blood loss (*p* = 0.002), and duration of hospital stay (*p* = 0.040) between the two groups. In the DCMS group, the mean duration of surgery was 210.8 ± 28.9 min, estimated blood loss was 814.5 ± 148.2 mL, and duration of hospital stay was 9.8 ± 2.5 days. Conversely, in the TCT group, these metrics were 252.6 ± 35.3 min, 927.4 ± 165.9 mL, and 11.2 ± 3.2 days, respectively. The differences observed in these three aspects between the two groups were statistically significant (Table 1).

**TABLE 1:** Comparison of patient characteristics between the two groups

	DCMS (*n* = 34)	TCT (*n* = 42)	Statistical value	*p*‐value
Age at surgery (years)	16.2 ± 3.8	15.4 ± 2.5	1.102	0.274
Sex (male/female)	8/26	10/32	0.001	0.977
Etiology (idiopathic/ neuromuscular)	29/5	33/9	0.565	0.452
Spinal cord classification (Type 1/2/3)	9/14/11	9/20/13	0.824	0.388
Risser sign	4.3 ± 0.5	4.2 ± 0.3	1.079	0.284
Flexibility of the main thoracic curve (%)	17.8 ± 5.2	19.4 ± 6.8	1.130	0.262
Instrumented levels	12.7 ± 2.3	12.4 ± 2.8	0.502	0.617
Number of screws	17.8 ± 3.7	16.9 ± 4.1	0.994	0.324
Duration of surgery (min)	210.8 ± 28.9	252.6 ± 35.3	5.558	**<0.001**
Estimated blood loss (mL)	814.5 ± 148.2	927.4 ± 165.9	3.092	**0.002**
Duration of hospital stay (days)	9.8 ± 2.5	11.2 ± 3.2	2.086	**0.040**
Follow‐up times (years)	3.2 ± 0.4	3.4 ± 0.7	1.481	0.143

*Note*: The bold values represent a statistically significant difference.

Abbreviations: DCMS, deformity correction manipulation system; TCT, Traditional correction techniques.

### Comparison of Radiological Outcomes and SRS‐30 Scores between DCMS and TCT

Regarding radiographic parameters (Table 2), the preoperative major curve of 82.4° ± 9.8° in the DCMS group was corrected to 20.7° ± 4.9° and 22.5° ± 6.7° at the 3‐month postoperative and final follow‐up, corresponding to a scoliosis correction of 74.2% ± 8.8%. In comparison, the preoperative major curve of 80.6° ± 10.2° (*p* > 0.05) in the TCT group was corrected to 23.3° ± 5.4° (*p* = 0.033) and 25.8° ± 6.1° (*p* = 0.028) at the 3‐month postoperative and final follow‐up, representing a scoliosis correction of 68.1% ± 10.5% (*p* = 0.009). While the two groups exhibited statistically similar preoperative major curves, the DCMS group achieved superior correction compared with the TCT group.

**TABLE 2:** Comparison of radiographic data between the two groups

	DCMS (*n* = 34)	TCT (*n* = 42)	Statistical value	*p*‐value
Cobb angle of the major curve (°)				
Preoperative	82.4 ± 9.8	80.6 ± 10.2	0.778	0.439
3‐month postoperative	20.7 ± 4.9	23.3 ± 5.4	2.174	**0.033**
Final follow‐up	22.5 ± 6.7	25.8 ± 6.1	2.244	**0.028**
Correction rate (%)	74.2 ± 8.8	68.1 ± 10.5	2.704	**0.009**
Thoracic kyphosis of T5‐T12 (°)				
Preoperative	30.2 ± 10.2	28.1 ± 12.2	0.802	0.425
3‐month postoperative	27.9 ± 5.4	27.4 ± 6.3	0.366	0.715
Final follow‐up	27.2 ± 4.7	26.6 ± 5.1	0.528	0.599
Lumbar lordosis of L1‐S1 (°)				
Preoperative	47.5 ± 12.6	46.3 ± 11.4	0.435	0.665
3‐month Postoperative	45. 9 ± 4.8	43.8 ± 5.2	1.811	0.074
Final follow‐up	46.3 ± 5.6	45.1 ± 6.0	0.893	0.375
Coronal imbalance (mm)				
Preoperative	21.1 ± 8.6	22.4 ± 7.4	0.704	0.481
3‐month Postoperative	14.6 ± 5.3	15.4 ± 5.1	0.668	0.506
Final follow‐up	15.3 ± 4.5	15.9 ± 4.2	0.600	0.551
Sagittal imbalance (mm)				
Preoperative	17.2 ± 10.7	17.5 ± 11.3	0.118	0.907
Postoperative	16.3 ± 5.2	16.6 ± 4.7	0.264	0.793
Final follow‐up	14.9 ± 6.2	15.1 ± 5.4	0.150	0.881
Apical vertebral translation (mm)				
Preoperative	60.1 ± 9.9	57.7 ± 10.3	1.028	0.308
Postoperative	21.9 ± 4.8	20.2 ± 5.5	1.417	0.161
Final follow‐up	20.3 ± 4.1	19.4 ± 4.2	0.939	0.351

*Note*: The bold values represent a statistically significant difference.

Abbreviations: DCMS, deformity correction manipulation system; TCT, Traditional correction techniques.

In terms of thoracic kyphosis and lumbar lordosis, there were no statistically significant differences between the two groups (*p* > 0.05). In the DCMS group, preoperative thoracic kyphosis of 30.2° ± 10.2° was restored to 27.9° ± 5.4° at 3 months postoperative and 27.2° ± 4.7° at the final follow‐up. Similarly, preoperative lumbar lordosis of 47.5° ± 12.6° was restored to 45.9° ± 4.8° at 3 months postoperative and 46.3° ± 5.6° at the final follow‐up. In the TCT group, preoperative thoracic kyphosis of 28.1° ± 12.2° was restored to 27.4° ± 6.3° at 3 months postoperative and 26.6° ± 5.1° at the final follow‐up. Additionally, preoperative lumbar lordosis of 46.3° ± 11.4° was restored to 43.8° ± 5.2° at 3 months postoperative and 45.1° ± 6.0° at the final follow‐up.

**FIGURE 4:**
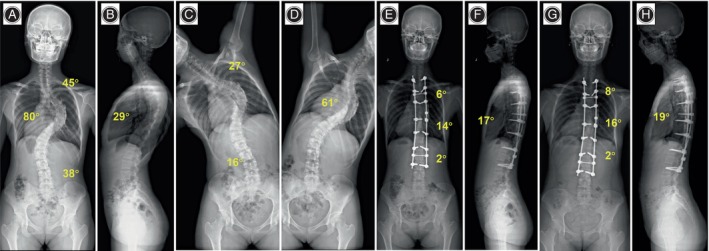
A 17‐year‐old girl with neuromuscular associated scoliosis in DCMS group. (A–D) Preoperative anteroposterior and lateral radiographs showed an 80° main thoracic curve, and the bending radiograph suggested a flexibility of 23.8% of the main curve. Thoracic kyphosis was 29°. (E, F) 3 months postoperative anteroposterior and lateral radiographs. The main thoracic curve was corrected to 14°, and the thoracic kyphosis was restored to 17°. The correction rate of the main curves was 82.5%. (G, H) Anteroposterior and lateral radiographs were taken at the 28‐month follow‐up. The main curve was corrected to 16° with a correction rate of 80.0%.

**FIGURE 5:**
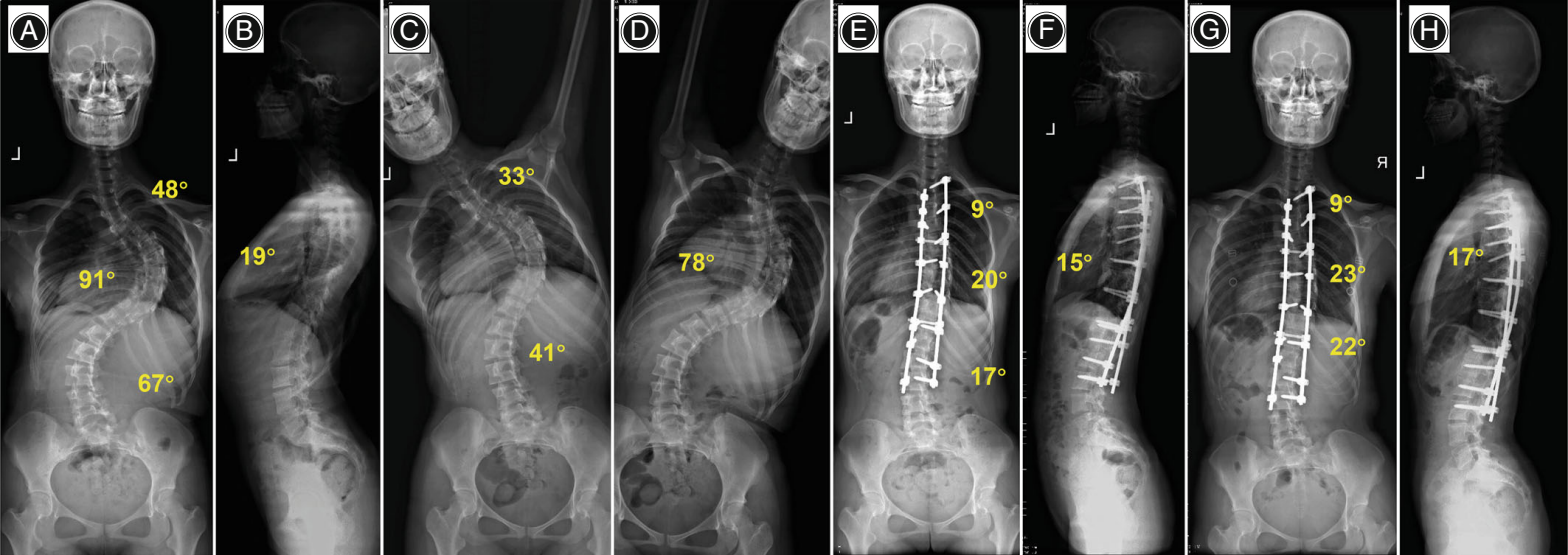
A 19‐year‐old girl with Lenke4CN idiopathic scoliosis in DCMS group. (A–D) Preoperative anteroposterior and lateral radiographs showed a 91° main thoracic curve, and the bending radiograph suggested a flexibility of 15.2% of the main curve. Thoracic kyphosis was 19°. (E, F) 3 months postoperative anteroposterior and lateral radiographs. The main thoracic curve was corrected to 20°, and the thoracic kyphosis was restored to 15°. The correction rate of the main curves was 78.0%. (G, H) Anteroposterior and lateral radiographs were taken at the 36‐month follow‐up. The main curve was corrected to 23° with a correction rate of 74.7%.

**FIGURE 6:**
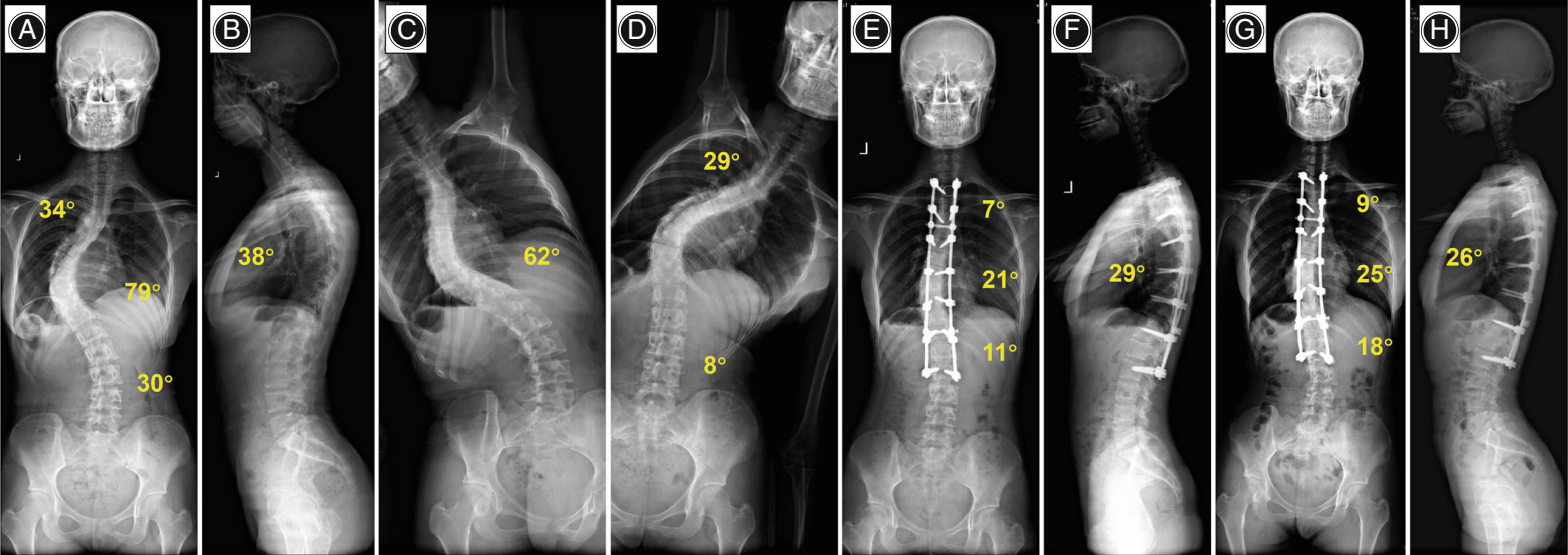
An 18‐year‐old girl with neuromuscular scoliosis in TCT group. (A–D) Preoperative anteroposterior and lateral radiographs showed a 79° main thoracic curve, and the bending radiograph suggested a flexibility of 21.5% of the main curve. Thoracic kyphosis was 38°. (E, F) 3 months postoperative anteroposterior and lateral radiographs. The main thoracic curve was corrected to 21°, and the thoracic kyphosis was restored to 29°. The correction rate of the main curves was 73.4%. (G, H) Anteroposterior and lateral radiographs were taken at the 26‐month follow‐up. The main curve was corrected to 25° with a correction rate of 68.4%.

**FIGURE 7:**
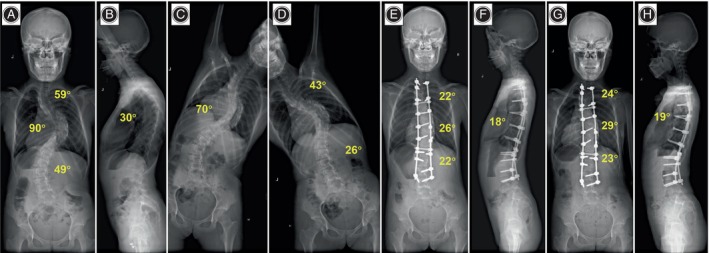
A 15‐year‐old girl with Lenke4CN idiopathic scoliosis in TCT group. (A–D) Preoperative anteroposterior and lateral radiographs showed a 90° main thoracic curve, and the bending radiograph suggested a flexibility of 22.2% of the main curve. Thoracic kyphosis was 30°. (E, F) 3 months postoperative anteroposterior and lateral radiographs. The main thoracic curve was corrected to 26°, and the thoracic kyphosis was restored to 18°. The correction rate of the main curves was 71.1%. (G, H) Anteroposterior and lateral radiographs were taken at the 24‐month follow‐up. The main curve was corrected to 29° with a correction rate of 67.8%.

Regarding coronal imbalance, sagittal imbalance, and apical vertebral translation, no significant differences (*p* > 0.05) were observed between the two groups. In the DCMS group, coronal imbalance improved from 21.1 ± 8.6 mm preoperatively to 14.6 ± 5.3 mm at 3 months postoperative and 15.3 ± 4.5 mm at the final follow‐up. Sagittal imbalance decreased from 17.2 ± 10.7 mm preoperatively to 16.3 ± 5.2 mm at 3 months postoperative and 14.9 ± 6.2 mm at the final follow‐up. Apical vertebral translation decreased from 60.1 ± 9.9 mm preoperatively to 21.9 ± 4.8 mm at 3 months postoperative and 20.3 ± 4.1 mm at the final follow‐up. Similarly, in the TCT group, coronal imbalance improved from 22.4 ± 7.4 mm preoperatively to 15.4 ± 5.1 mm at 3 months postoperative and 15.9 ± 4.2 mm at the final follow‐up. Sagittal imbalance decreased from 17.5 ± 11.3 mm preoperatively to 16.6 ± 4.7 mm at 3 months postoperative and 15.1 ± 5.4 mm at the final follow‐up. Apical vertebral translation decreased from 57.7 ± 10.3 mm preoperatively to 20.2 ± 5.5 mm at 3 months postoperative and 19.4 ± 4.2 mm at the final follow‐up. Typical cases in the DCMS group and TCT group were shown in Figures 4, 5, 6, 7, respectively.

**FIGURE 8:**
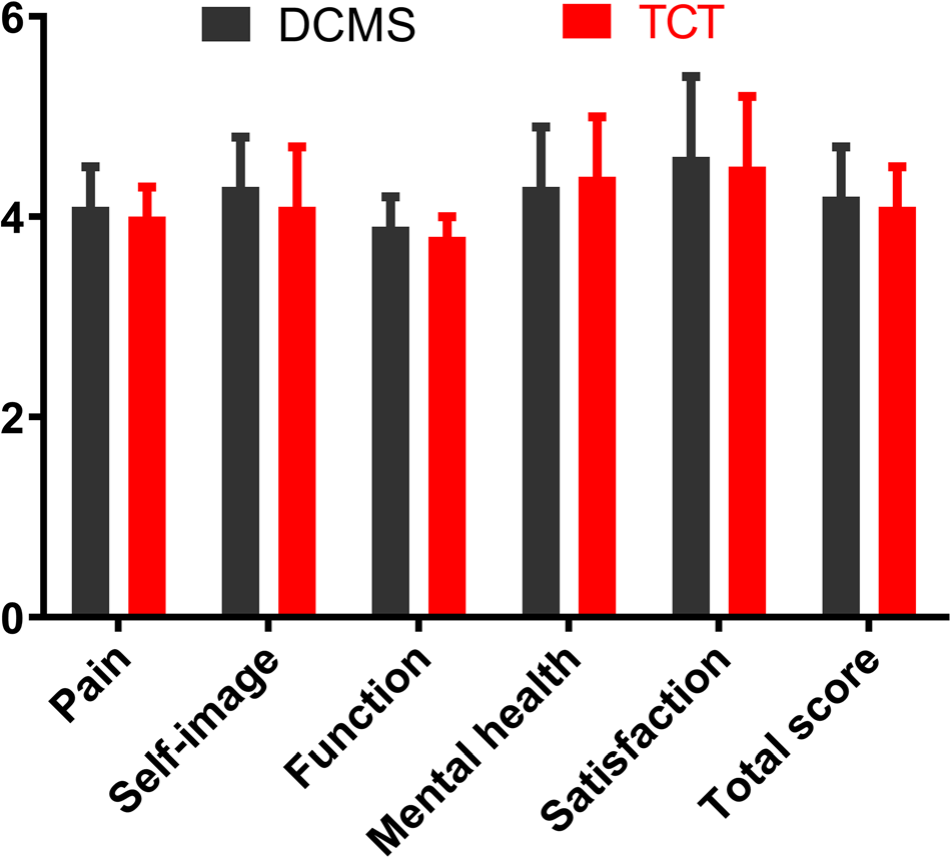
The comparison of SRS‐30 score in the patients with DCMS and TCT.

The final follow‐up SRS‐30 scores for the DCMS and TCT groups are depicted in Figure 8. Across all domains including pain, self‐image, function, mental health, and satisfaction, no significant differences were observed between the two groups (*p* > 0.05).

### Complications

Adverse events were documented in both groups, as outlined in Table 3. Intraoperative neuromonitoring changes during correction maneuvers were noted in four patients (one with Type 2 spinal cord and three with Type 3 spinal cord) in the DCMS group and two patients (both with Type 3 spinal cord) in the TCT group. Specifically, in the DCMS group, two patients experienced a 50% decrease in MEPs relative to peak value and recovered after 60 min; one patient experienced a 60% decrease in MEPs relative to peak value and did not recover until the end of surgery; and one patient experienced a 60% decrease in SSEPs and a 30% decrease in MEPs relative to peak and did not recover until the end of surgery. In the TCT group, one patient experienced a 50% decrease in MEPs relative to peak value and recovered after 30 min; and one patient experienced a 50% decrease in MEPs relative to peak value and did not recover until the end of surgery. Fortunately, no transient or permanent neurological deficits occurred in either group. Additionally, in the TCT group, two patients experienced pedicle fractures in the upper thoracic region, whereas no such occurrences were reported in the DCMS group. Furthermore, due to intraoperative blood loss, three patients in the DCMS group and six patients in the TCT group required blood transfusion support therapy. Notably, there were no cases of infection or pseudarthrosis reported in either group.

**TABLE 3:** Adverse events

	DCMS (*n* = 34)	TCT (*n* = 42)	Statistical value	*p*‐value
Neuromonitoring change	4 (11.8%)	2 (4.8%)	0.487	0.485
Pedicle fracture	0	2 (4.8%)	1.641	0.200
Blood transfusion	3 (8.8%)	6 (14.3%)	0.141	0.707
Neurologic deficit	0	0		/
Infection	0	0		/
Pseudarthrosis	0	0		/

Abbreviations: DCMS, deformity correction manipulation system; TCT, Traditional correction techniques.

## Discussion

Our research demonstrates that DCMS is an effective technique for treating large thoracic scoliosis. Compared to TCT, the use of DCMS significantly reduces blood loss and shortens operation time, benefiting patients by accelerating postoperative recovery and reducing hospital stays. Furthermore, DCMS enhances the application of corrective force, improving the deformity correction rate. The safety of DCMS has also been validated in this study, with a manageable and comparable incidence of complications to TCT.

### DCMS Performance

The design of the DCMS was inspired by the Posterior Rod‐Link‐Reducer System invented by Zhang *et al*.,^19^ as well as the cantilever bending technique pioneered by Chang.^20^ The DCMS capitalizes on the robust moment arms and provides powerful handles on the manipulation arm to facilitate the application of correction force. In contrast to the single screw anchoring points utilized in TCT, the DCMS offers multiple anchoring points for long segmental deformity correction. Restoring kyphosis is a critical objective in the treatment of thoracic scoliosis. Failure to adequately restore kyphosis may result in sagittal imbalance of the entire spine and may also be associated with postoperative back pain.^21^ The lifting force applied to periapical vertebrae during concave side distraction is specifically designed to restore thoracic kyphosis. Additionally, the provision of persistent and adjustable distraction or compression force throughout the procedure helps to maintain the degree of correction, reduce correction loss, and prevent rod bounce back. Importantly, the DCMS operation is straightforward and reduces the complexity of the surgical procedure.

### Efficacy of DMCS

In this retrospective study, the utilization of DCMS demonstrated shorter operative times, reduced blood loss, and shorter hospital stays compared to TCT. Excessive bleeding and prolonged anesthesia time are highlighted as factors contributing to perioperative complications.^22^, ^23^, ^24^ For instance, prolonged hypotension resulting from significant blood loss during surgery poses a risk factor for neurological complications.^25^ The shorter hospital stays associated with DCMS signify faster patient recovery and potentially lower treatment costs. Our radiographic data indicate that DCMS provides superior correction of the major curve and similar restoration of sagittal balance compared to TCT. The effectiveness of the correction force generated by DCMS has been validated. However, there were no significant differences observed in patient self‐reported outcomes. This discrepancy between radiographic data and cosmetic appearance may suggest that solely achieving better major curve correction might not suffice to elicit a minimum clinically important difference.^26^

### Complications and Technical Experience

Interestingly, five of the six patients who experienced intraoperative neuromonitoring changes had a Type 3 spinal cord. Type 3 spinal cords may be less resilient to reduced blood supply during deformity correction, and this characteristic could serve as an indicator of the risk of having intraoperative neuromonitoring alerts. Certain strategies should be adopted for these patients to mitigate these risks: (i) a thorough preoperative radiographic assessment is necessary to identify the high‐risk patients and determine spinal cord compression; (ii) systemic optimization and augmentation of mean arterial pressure and hematocrit should be performed during surgery; (iii) stabilization of the spinal column should be ensured as much as we can to reduce spinal cord disturbance; and (iv) distraction and compression force should be performed slowly.^27^, ^28^ It is noteworthy that there is a higher likelihood of intraoperative neuromonitoring changes with the use of DCMS. The strong force easily generated by DCMS sometimes leads to a significant correction occurring rapidly, potentially causing spinal cord interference. This may explain the observed neuromonitoring changes in the DCMS group. This suggests that for such patients, a slow and gradual correction with controllable and adjustable force is crucial for Type 3 spinal cord patients. Fortunately, no patients reported neurological deficits. Moreover, it is important to consider the maldevelopment of vertebrae in scoliosis, particularly in the proximal thoracic and concave apical regions, which often have narrow and weak pedicles.^29^, ^30^ These areas are prone to pedicle fractures when subjected to significant stress during deformity correction procedures. The DCMS may offer more dispersive stress distribution, potentially reducing the likelihood of pedicle fractures. Notably, skip pedicle screw instrumentation with low‐density pedicle screws was performed on our patients, a technique previously shown to achieve good clinical outcomes comparable to all‐pedicle screw instrumentation while reducing screw‐related complications.^31^, ^32^ In cases where pedicle fractures occurred in the TCT group, alternative pedicles could be selected for screw fixation. Although no significant difference was observed in the incidence of adverse events between the two methods, this may be attributed to the small number of patients studied. Further research on a larger scale is warranted to validate the effectiveness and safety of the DCMS technique.

### Strengths and Limitations

Several limitations should be acknowledged in this study. First, it is important to note that our study was conducted at a single center and involved a relatively small sample size, which may introduce bias into the results. Second, due to the retrospective design of the study, it is uncertain as to the surgeons' intentions regarding deformity correction, which could potentially influence the outcomes. Last, considering the risk of radiation exposure, routine postoperative chest computed tomography examinations were not performed, preventing the analysis of thoracic morphology between the two surgical techniques. Future research with larger sample sizes and prospective designs is needed to address these limitations and further validate the outcomes.

### Prospects of Clinical Application

DCMS offers a relatively straightforward yet effective technique for the treatment of large thoracic scoliosis. A comprehensive preoperative assessment is essential to evaluate the status of the spinal cord in these patients. Special caution is warranted when using DCMS in patients with spinal cord compression or deformation.

## Conclusion

In a retrospective cohort study involving patients with large thoracic scoliosis, the utilization of the DCMS demonstrated several advantages over the traditional correction techniques (TCT) group. Specifically, patients treated with DCMS exhibited shorter operative times, reduced blood loss, shorter hospital stays, and greater curve correction compared with those in the TCT group. These findings suggest that DCMS offers a safe and effective approach for achieving improved correction of large thoracic curves without introducing additional surgical risks.

## Author Contributions

YH and CZ conceptualized and designed the study, drafted the initial manuscript. YW, JW, RW and XY carried out the initial analyses, reviewed and revised the manuscript. GF, and LL coordinated and supervised data collection, critically reviewed and revised the manuscript for important intellectual content. All authors approved the final manuscript as submitted and agree to be accountable for all aspects of the work.

## Conflict of Interest Statement

The authors declare that they have no competing interests.

## Ethics Statement

This study was approved by the ethics committee of West China Hospital of Sichuan University and informed consent was obtained from the patients.

## Consent for Publication

The patients gave written consent for publication of their clinical details and clinical images. A copy of the written consent is available for review from the editor of this journal.

## Supporting information


Video S1.

Video S2.

## Data Availability

Data will be available upon request to the corresponding author.
